# Social support: an important factor for quality of life in women with hirsutism

**DOI:** 10.1186/s12955-014-0183-3

**Published:** 2014-12-20

**Authors:** Maria Palmetun Ekbäck, Magnus Lindberg, Eva Benzein, Kristofer Årestedt

**Affiliations:** Department of Dermatology, University Hospital Örebro, 701 85 Örebro, Sweden; Faculty of Medicine and Health, Örebro University, SE 70182 Örebro, Sweden; Department of Pharmacology and Therapeutics, Örebro County Council, Örebro, Sweden; Department of Health and Caring Science, Linnaeus University, Kalmar, Sweden; Department of Medical and Health Sciences, Linköping University, Linköping, Sweden; Palliative Research Centre, Ersta Sköndal University College and Ersta Hospital, Stockholm, Sweden

**Keywords:** Association, Health, Hirsutism, Quality of life, Social support

## Abstract

**Background:**

Women with hirsutism have reported imparied health and health-related quality of life (HRQoL). Social support is a factor that might increase HRQoL in chronic diseases, but little is known about this association among women with hirsutism.

**Aim:**

The aim of the study was to describe social support and explore its association with HRQoL among women with hirsutism. A further aim was to compare HRQoL in women with hirsutism with a Swedish normal population.

**Methods:**

A questionnaire including socio-demographic questions, Short-Form Health Survey (SF-36), the Multidimensional Scale of Perceived Social Support (MSPSS), and a self-estimation of hairiness using the Ferriman-Gallway scale (F-G scale) was answered by 127 women with hirsutism.

**Results:**

Multiple regression analyses showed significant associations between social support and all health dimensions in the SF-36, also after the model was adjusted for age, hairiness and body mass index. Compared to the normal Swedish population, women with hirsutism reported significantly lower HRQoL in all dimensions of the SF-36 (p < 0.01).

**Conclusions:**

There is a significant positive association between social support and HRQoL, demonstrating its importance for the ability to adapt to problems associated with hirsutism. As women with hirsutism reported poorer HRQoL compared to the normal population, social support may be a factor to consider in clinical practice.

## Background

It is known that chronic skin diseases can be accompanied by psychological and social disabilities [1-3]. One such disease is hirsutism, which means a woman having excessive terminal hair in a male pattern distribution. It is caused by elevated androgen exposure to the hair follicles [4-6], mostly because of polycystic ovary syndrome (PCOS) [7], or by an increased sensitivity to normal levels of circulating androgens, i.e., idiopathic hirsutism. Hirsutism has been reported to have a negative effect on health-related quality of life (HRQoL) [8-11], and anxiety and depression is more common than among women without hirsutism [12]. In women with PCOS, changes due to PCOS might contribute to psychological morbidity [10,13-19] and a feeling of being stigmatized [20].

Adaptation to living with a chronic disease encompasses a range of phenomena, e.g., HRQoL, emotional well-being, acceptance of a life with a chronic disease and finding a way of participating in society [21]. Social support could be one contributor to the adaption to a life with a chronic disease and a factor that affects the outcome of HRQoL. Furthermore, it is known to influence outcomes such as anxiety and depression in a variety of chronic diseases [22-24]. Social support is commonly used as a general concept that includes some kind of relationship transactions between individuals. Perceived social support seems to be most important [25] and can be emotional, instrumental (practical), informative, or appraisal [26]. It could be that the quality of the social support among women with hirsutism determines how well they can cope with their situation and adapt in social situations.

The association between social support and HRQoL in skin diseases has only been studied to a minor extent [21,27], and to our knowledge, not at all in women with hirsutism. The aim of the present study was therefore to describe social support and explore its association with HRQoL among women with hirsutism. A further aim was to compare HRQoL in women with hirsutism with a Swedish normal population.

## Methods

### Sample

Women who, according to their medical records, were diagnosed with hirsutism at the departments of dermatology in Malmö (n = 100) and Örebro (n = 80), or had on-going treatment for hirsutism (n = 20) at a private dermatological clinic in Uppsala were invited to participate in the study. A questionnaire was sent with one reminder to the 200 identified women, of whom 132 responded (66%). Five women were excluded because of wrong diagnosis (which was found in the questionnaire), psychiatric illness or failure to complete the questionnaires. The study sample finally consisted of 127 women. It was possible to identify 55 of the non-respondents. Reasons given for not participating were: difficult language in the questionnaire (n = 11), forgotten to answer (n = 11), too private questions (n = 15), did not have time to fill in the questionnaire (n = 5), no problems anymore (n = 4), do not want to answer (n = 1), and no reason given (n = 8).

A Swedish normal sample was recruited from a nationwide survey based on a questionnaire on subjectively perceived health, quality of life (SF-36), and use of medication [28]. Sweden keeps a population register based on the unique personal number held by all people who are legal residents in the country. The questionnaire was sent by mail to a random sample from this register, i.e., a random sample of the Swedish population (n = 7985), aged 18–84 years. The survey was performed between October 2004 and January 2005. We obtained the SF-36 data for an age-adjusted sample of 1 115 women from this survey.

The study was conducted between October 2010 and August 2012. It was approved by the Regional Ethics Committee in Uppsala (study code: 2010/207).

### Data collection and measures

Data were collected by a self-rated questionnaire, composed of 4 parts; *i)* socio-demographic questions (age, employment, education, civil status, children, body mass index (BMI), income, satisfaction with work, social contact), *ii)* self-estimation of hairiness (the Ferriman-Gallwey scale, F-G scale), *iii)* HRQoL (the 36 Short- Form Health Survey, SF-36) and *iv)* perceived social support (the Multidimensional Scale of Perceived Social Support, MSPSS).

#### The Ferriman-Gallwey scale

The modified Ferriman-Gallwey scale (F-G scale) is a visual self-rating method to determine hairiness in nine androgen dependent body areas [29,30]. The scale ranges from 0 to 36, and the cut-off score for hirsutism is a value over six, except in facial areas where a F-G score ≥ 2 also is considered as hirsutism [31,32].

#### The 36 short-form health survey

The 36 Short- Form Health Survey (SF-36) is a generic HRQoL instrument. It contains 36 items and eight health domains: physical functioning (PF, limitation in performing physical activities such as bathing or dressing), role-physical (RP, limitation in work and other daily activities), bodily pain (BP, bodily pain), general health (GH, how the person perceives her/his own general health), vitality (VT, a feeling of being tired, worn out vs. feeling energetic), social functioning (SF, interference with normal social activities due to physical or emotional problems), role emotional (RE, limitations in daily activities or work due to emotional problems), and mental health (MH, feeling happy or calm vs. nervous and depressed). Scores for each health domain range from 0 to 100. The higher the score, the better the health in that domain. The questionnaire has two main components, one physical (PCS) and one mental (MSC), also with a possible score range between 0 and 100.

#### The multidimensional scale of perceived social support

The Multidimensional Scale of Perceived Social Support (MSPSS) was constructed by Zimet et al. [25] and aims to assess perceived social support. It includes 12 items which cover three dimensions: family, friends and significant others. Each item is rated on a seven-point Likert-type scale (1 = very strongly disagree; 7 = very strongly agree). A total score is calculated by summing the results for all items. Scores range between 12 and 84, where higher scores indicate higher perceived social support. In addition, separate subscales can be used by summing the responses from the items in each of the three dimensions. The possible score range for the subscales is 4 to 28 respectively. The instrument has been found to be reliable and valid, both in its original language [33] and in other languages [34,35]. In the present study, a Swedish validated version of the MSPSS was used [36].

### Statistical analysis

Descriptive statistics were used to describe patient demographics, clinic characteristics, perceived social support, and HRQoL.

The association between social support and HRQoL was explored by multiple linear regression analyses [37]. In a first step (the initial model), HRQoL were entered as the outcome variable, while social support was entered as the independent variable. In a second step (the full model), age, self-reported hairiness, and BMI were added as adjusting covariates. As the subscales of social support were highly correlated with one another (i.e. Family, Friends and Significant others), the MSPSS total score was used to avoid problems with multicolinearity.

Independent sample *t*-test was used to compare the SF-36 dimensions between the study sample and the normal population sample [38]. Cohen’s *d* effect size was used to estimate the clinical importance of the difference between the two groups [39]. The interpretation of Cohen’s *d* effect size was as following: small (0.2), medium (0.5), or large (0.8) [40].

The level of statistical significance was set at *p* < 0.05. All analyses were conducted using SPSS version 21 for Windows (IBM Corp, Armonk, NY, USA).

## Results

### Charateristics of the participants

The characteristics of the women with hirsutism and the Swedish normal population sample are given in Table 1. The mean age of women with hirsutism was 32.0 years. Among the respondents, 44.9% lived with a partner, 48.8% had children living at home, and 55.1% of the women were working. Half of the women (53%) were satisfied with the contact they had with family, friends, and colleagues, while the others wanted more contact.

**Table 1:** **Characteristics of the participants**

	**Study sample, n = 127**	**Normal population sample, n = 1115**
Age, mean years (SD)	32.0 (10.2)	32.7 (7.9)
Gainfully employed, n (%)		
Full time	55 (43.3)	
Part time	15 (11.8)	
Not working	35 (27.6)	
Unknown	22 (17.3)	
Civil status, n (%)		
Living with a partner	57 (44.9)	
Living alone	65 (51.2)	
Unknown	5 (3.9)	
Children at home, n (%)		
Having children living at home	62 (48.8)	
Having not children living at home	65 (51.2)	
BMI, mean (SD) [range]	28.2 (6.4) [17.8 - 47.7]	
F-G score, mean (SD)	18.8 (8.4)	
MSPSS, mean (SD)		
Total	62.5 (18.2)	
Family	21.2 (7.0)	
Friends	18.5 (8.0)	
Significant other	22.7 (6.7)	

BMI = Body Mass Index, F-G = Ferriman Gallwey score, MSPSS = Multidimensional Scale of Perceived Social Support.

The normal population sample consisted of 1 115 women with a mean age of 32.7 years. There were no significant age differences between the women with hirsutism and the normal population sample (*p* = 0.416).

### Social support and the association with HRQoL

According to the distribution of MSPSS scores, women with hirsutism reported generally high levels of perceived social support, but with great individual variations (Table 1). Perceived social support measured with MSPSS revealed a mean score of 62.5. Of the three subscales, the highest mean score was found in the subscale Significant others (mean = 22.7). A mean score of 21.2 was reported for the Family subscale, while the Friends subscale had the lowest mean score (mean = 18.5). Scores lower than 15 were reported by 33% of the women on the Friends subscale, by 19% on the Family subscale, and by 13% on the Significant others subscale.

The multiple linear regression analysis revealed a significant association between perceived social support and HRQoL (Table 2). In the initial model, social support (MSPSS total score) was significantly associated with all health domains in the SF-36, as well as the MCS. Social support explained 6% to 34% of the total variance in the health domains in the SF-36 and 30% of the total variance in the MCS. However, social support explained a larger share of the total variance in the mental domains (Vitality, Social Functioning, Role Emotional and Mental Health, *R*^2^ = 0.13-0.34), compared to the physical domains (Physical Functioning, Role Physical, Bodily Pain and General Health, *R*^2^ = 0.06-0.12). The association between social support and HRQoL also remained after including the adjusting covariates in the full model. The full model, including all independent variables explained 14% (Vitality) to 37% (Mental Health and MCS) of the total variance in the different health domains and component summary scores.

**Table 2:** **Associations between social support and health-related quality of life**

		**Initial model**		**Full model**	
Dependent variable	Independent variables	β (SE)	95% CI for β	β (SE)	95% CI for β
Physical Functioning	MSPSS	0.36 (0.10)^***^	0.15/0.56	0.26 (0.10)^**^	0.07/0.45
n = 123	Age			−0.72 (0.18)^***^	−1.07 / -0.38
	F-G score			−0.35 (0,21)	−0.77/0.07
	BMI			−0.81 (0.27)^**^	−1.34/-0.28
	Model statistics:	F(1, 121) = 12.10, p < 0.001, R^2^ = 0.09		F(4, 120) = 11.31, p < 0.001, R^2^ = 0.29	
Role-Physical	MSPSS	0.70 (0.17)^***^	0.40/1.08	0.63 (0.18)^***^	0.28/0.98
n = 123	Age			−0.47 (0.32)	−1.12/0.17
	F-G score			−0.55 (0.40)	−1.34/0.24
	BMI			−0.70 (0.51)	- 1.71/0.32
	Model statistics	F(1, 121) = 18.50, p < 0.001, R^2^ = 0.13		F(4, 118) = 6.26, p < 0.001, R^2^ = 0.18	
Bodily Pain	MSPSS	0.43 (0.14)^**^	0.14/0.71	0.28 (0.14)^*^	0.02/0.56
n = 125	Age			−15.47 (5.15)^***^	−1.37/-0.36
	F-G score			−0.61 (0.30)^*^	−1.33/-0.11
	BMI			- 0.97 (0.39)^*^	−1.66/-0.11
	Modell statistics	F(1, 123) = 8.85, p = 0.004, R^2^ = 0.06		F(4, 120) = 8.43, p < 0.001, R^2^ = 0.19	
General Health	MSPSS	0.46 (0.11)^***^	0.24/0.68	0.38 (0.11)^***^	0.16/0.60
n = 125	Age			−0.13 (0.21)	−0.54/0.27
	F-G score			−0.17 (0.25)	−0.66/0.32
	BMI			−1.22 (0.32)^***^	−1.84/-0.59
	Model statistics	F(1, 123) = 16.54, p < 0.001,R^2^ = 0.12		F(4, 120) = 9.09, p < 0.001, R^2^ = 0.23	
Vitality	MSPSS	0.46 (0.11)^***^	0.26/0.67	0.41 (0.11)^***^	0.20/0.63
n = 125	Age			0.08 (0.20)	−0.31/0.48
	F-G score			−0.35 (0.24)	−0.82/0.13
	BMI			−0.37 (0.31)	−0.97/0.24
	Model statistics	F(1, 123) = 19.48, p < 0.001, R^2^ = 0.13		F(4, 120) = 6.01, p < 0.001,R^2^ = 0.14	
Social Functioning	MSPSS	0.84 (0.14)^***^	0.55/1.12	0.70 (0.14)^***^	0.42/0.98
n = 125	Age			0.13 (0.26)	−0.38/0.64
	F-G score			−1.24 (0.31)^***^	−1.86/-0.62
	BMI			−0.32 (0.40)	- 1.11/0.47
	Model statistics	F(1, 123) = 33.48, p < 0.001, R^2^ = 0.21		F(4, 120) = 14.56, p < 0.001, R^2^ = 0.33	
Role-Emotional	MSPSS	1.15 (0.19)^***^	0.77/1.53	1.07 (0.20)^***^	0.67/1.47
n = 122	Age			0.04 (0.36)	−0.68/0.76
	F-G score			−0.56 (0.45)	−1.44/0.33
	BMI			−0.43 (0.58)	−1.57/0.71
	Model statistics	F(1, 120) = 35.77, p < 0.001, R^2^ = 0.23		F(4, 117) = 9.50, p < 0.001, R^2^ = 0.25	
Mental Health	MSPSS	0.79 (0.10)^***^	0.59/0.98	0.74 (0.10)^***^	0.54/0.94
n = 125	Age			0.21 (0.18)	−0.15/0.57
	F-G score			−0.48 (0.22)^*^	−0.92/-0.04
	BMI			−0.09 (0.28)	−0.65/0.47
	Model statistics	F(1, 123) = 64.79, p < 0.001, R^2^ = 0.34		F(4,120) = 19.14, p < 0.001, R^2^ = 0.37	
PCS	MSPSS	0.10 (0.06)	−0.06/0.21	0.05 (0.05)	−0.05/0.15
n = 122	Age			−0.37 (0.09)^***^	−0.55/-0.19
	F-G score			−0.16 (0.11)	−0.38/0.07
	BMI			−0.48 (0.15)^***^	−0.77/-0.19
	Model statistics	F(1, 120) = 3.51, p = 0.064, R^2^ = 0.03		F(4, 117) = 9.84, p < 0.001, R^2^ = 0.34	
MCS	MSPSS	0.44 (0.06)^***^		0.41 (0.06)^***^	0.29/0.54
n = 122	Age			0.23 (0.11)^*^	0.01/0.45
	F-G score			−0.33 (0.14)^*^	−0.61/-0.05
	BMI			−0.05 (0.18)	−0.40/0.31
	Model statistics	F(1, 120) = 49.91, p < 0.001, R^2^ = 0.30		F(4, 117) = 17.36, p < 0.001, R^2^ = 0.37	

*p < 0.05; **p < 0.01; ***p < 0.001.

MSPSS = Multidimensional Scale of Perceived Social Support, PCS = Physical Component Summary, MCS = Mental Component Summary, BMI = Body Mass Index.

Among the covariates, hairiness was significantly associated with bodily pain, social functioning, mental health, and MCS. Age and BMI were both associated with physical functioning bodily pain and PCS. In addition, age was also associated with MCS, while BMI was associated with general health.

### HRQoL comparison

**Figure 1:**
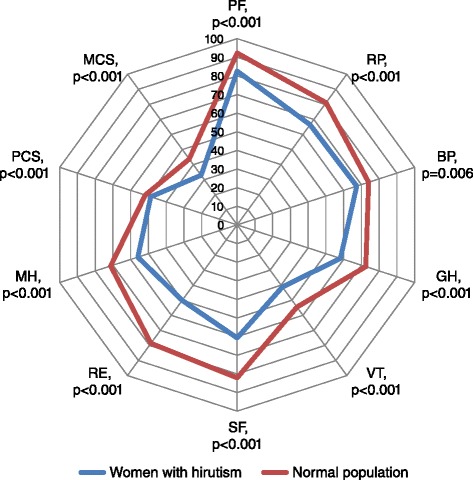
**Health-related quality of life comparisons between women with hirutism and the Swedish normal population.** PF = Physical Functioning, RP = Role Physical, BP = Bodily Pain, GH = General Health, VT = vitality, SF = Social Functioning, RE = Role Emotional, MH = Mental Health, PCS = Physical Component Summary, MCS = Mental Component Summary.

Women with hirsutism and the normal population sample both reported greatest health in Physical Functioning, 82.6 and 92.3 respectively. Both women with hirsutism and the normal population sample reported poorest health in Vitality, 41.2 and 54.5 respectively. Women with hirsutism reported significantly lower HRQoL than women in the normal population sample in each health domain as well as PCS and MCS (Table 3 & Figure 1). The effect size was medium or large for all health domains, except Role Physical (*d* = 0.42), Bodily Pain (*d* = 0.24), and PCS (*d* = 0.31). The largest effect size between the two groups was shown for Social Functioning (*d* = 0.75), Role Emotional (*d* = 0.72), and MCS (*d* = 0.79). With the exception of General Health (*d* = 0.61), the largest effect size was demonstrated for the emotional domains and MCS (Table 3).

**Table 3:** **Health-related quality of life comparisons between women with hirsutism and the Swedish normal population**

**SF-36 subscales**	**Hirsutism mean (SD)**	**n**	**Normal population mean (SD)**	**n**	**p-value** ^**a**^	**ES** ^**b**^
Physical Functioning	82.6 (21.5)	126	92.3 (14.3)	1113	<0.001	0.53
Role-Physical	66.5 (36.8)	124	81.1 (32.7)	1115	<0.001	0.42
Bodily Pain	67.4 (29.8)	126	74.1 (25.8)	1113	0.006	0.24
General Health	58.2 (24.2)	126	72.5 (22.4)	1105	<0.001	0.61
Vitality	41.2 (22.7)	126	54.5 (23.1)	1113	<0.001	0.58
Social Functioning	60.7 (32.8)	126	82.1 (23.4)	1114	<0.001	0.75
Role-Emotional	50.1 (43.4)	123	78.6 (35.1)	1111	<0.001	0.72
Mental Health	56.0 (24.2)	126	71.3 (20.1)	1111	<0.001	0.69
PCS	48.7 (11.0)	123	51.8 (9.0)	1105	<0.001	0.31
MCS	32.9 (14.8)	123	43.7 (12.4)	1105	<0.001	0.79

^a^Independent sample t-test.

^b^Cohen’s d effect size: 0.2 = small, 0.5 = medium, 0.8 = large.

A within group analysis comparing PCS with MCS showed that both women with hirsutism and the normal population sample reported significantly poorer mental health compared to physical health, t(122) = 9.50, *p* < 0.001 and t(1104) = 17.57, *p* < 0.001 respectively. However, the effect size was smaller in the normal population sample compared with women with hirsutism, *d* = 0.75 and *d* =1.21 respectively.

## Discussion

To the best of our knowledge, this is the first study to explorethe association between social support and HRQoL in women with hirsutism. The main findings show that social support is significantly associated with HRQoL, and shown to be most prominent with mental health, but also associated with physical health. In the present study, we also demonstrate that HRQoL was significantly lower in all measured health domains in women with hirsutism compared to a normal population of Swedish women.

The significant association between social support and HRQoL indicates that the importance of family and friends as supporters cannot be underestimated in the care of women with hirsutism. Our findings are congruent with a qualitative study by our research group, where the women emphasised the importance of support from their family members and friends [9]. Picardi et al. [27] found that in psoriasis exacerbations, lack of social support affected not only the HRQoL outcome, but may also precede a psoriasis flare- up. A study of patients with breast cancer indicated that hopelessness among the patients, measured with Beck Hopelessness Scale (BH), was decreased when their social support increased [41].

The level of perceived social support measured with MSPSS in the present study was at similar levels to what has been found in patients with diffuse plaque psoriasis [27], but lower than in a Swedish group of nursing students dominated by younger women whithout visible skin diseases [36]. The reason for this could be that the women with hirsutism, having visible hair on their faces, feel socially vulnerable and stigmatized [20], as patients with visible psoriasis do [42], and therefore withdraw from social life.

In accordance with the findings in the present study, it has previously been reported that hirsutism has a negative effect on HRQoL [9]. Even if there are several options to reduce hair in women with hirsutism, restoring a body to normal hair growth is not possible. It is therefore important to identify and establish other factors, e.g., social support, that can be part of the treatment and care for these women. There are different types of social support, one being informative support [26]. Informative support is, for instance, provision of disease or health- relevant information to the patient and should be provided by healthcare personnel. It has been shown that in psoriasis, social support is generally more effective for preventing depression in women than in men [21]. It has also been shown that given a symptom, a diagnosis and information about that diagnosis brings relief to the patient [43]. It is therefore important that when seeking healthcare, women with hirsutism receive information and support [44].

From a socio-economic point of view, 55% of the women with hirsutism were gainfully employed, which is a lower proportion compared to the figure reported for the general female population in Sweden [45]. As for HRQoL, the results of this study demonstrate that all dimensions in the SF-36 were significantly lower compared to a Swedish normal population sample, but in accordance with patients with severe dermatological states, such as severe psoriasis [46], severe hand eczema [28] and ichtyosis [47]. The dimensions with the most impaired health were Vitality, Social Functioning, Role Emotional and Mental Health. The mean score for Vitality among hirsute women was even lower than reported in patients with myasthenia gravis and multiple sclerosis [48]. The dimensions with the greatest difference, according to the Cohen’s *d* effect size between the group of women with hirsutism and the normal population sample, was Social Functioning and Role Emotional, which further points to the fact that the women with hirsutism feel stigmatized [20].

### Methodological considerations

We have applied well established and validated techniques to obtain self-reported data from women with hirsutism. The use of the generic instrument SF-36 allows comparisons between different disease groups and general populations [49,50]. One limitation is that SF-36 is not specifically developed for measuring health in patients with skin problems. Dermatology- specific instruments, on the other hand, are more suited for skin diseases and as such allow comparisons between different skin diseases, but could not be used for comparison with other non-dermatological diseases. However, the SF-36 has been widely used with different objectives in patients with dermatology diseases. It has also been used as a reference tool for HRQoL in validation studies of a dermatology-specific instrument [51]. It has been compared with the Dermatology Life Quality Index (DLQI), by using factor analysis and found to be a useful instrument in measuring HRQoL in patients with hand eczema [1].

One limitation is that the patients may have been misdiagnosed as they were recruited from medical records. However, by using the F-G scale to define hairiness and hirsutism, the possible negative effects were reduced. Another limitation could be that we have recruited patients from three different centres, including one private clinic. However, all women included in the study were diagnosed with hirsutism by dermatologists, and the F-G scale was used to define hirsutism and location of hairiness. A further limitation is that this study had a cross-sectional design, which reduces the possibility to draw any causal conclusions about the relationship between social support and HRQoL. Longitudinal studies of social support and HRQoL are therefore needed.

No a priori sample size calculation was conducted, but according to most rules of thumb [52], the sample size can be deemed to be sufficiently large for a regression model including four independent varaibles. In addition, a post hoc power calculation showed that the statistical power (1- β) in the present study varied between 0.55 and 0.87 for the regression models. Despite a somewhat low power for some of the models, the association between social support and HRQoL was confirmed.

## Conclusions

In the present study, we have confirmed that hirsutism has a pronounced negative effect on women’s HRQoL, particularly their mental health. Interventions to improve HRQoL among women with hirustism therefore seem to be of importance. We found a significant association between perceived social support and HRQoL, indicating that improved social support is an important factor for enhancing HRQoL in hirsutism, particularly mental health but also physical health. In order to improve HRQoL, social support in clinical practice therefore seems to be a factor to consider.
